# Cultural Sensitivity in Interventions Aiming to Reduce or Prevent Intimate Partner Violence During Pregnancy: A Scoping Review

**DOI:** 10.1177/15248380211021788

**Published:** 2021-06-10

**Authors:** Lena Henriksen, Sezer Kisa, Mirjam Lukasse, Eva Marie Flaathen, Berit Mortensen, Elisabeth Karlsen, Lisa Garnweidner-Holme

**Affiliations:** 1 Department of Nursing and Health Promotion, Oslo Metropolitan University, Norway; 2 Division of General Gynaecology and Obstetrics, Oslo University Hospital, Norway; 3 Department of Nursing and Health Sciences, University of South-Eastern Norway, Kongsberg, Norway

**Keywords:** intimate partner violence, pregnant women, pregnancy, interventions, cultural sensitivity, culture

## Abstract

Intimate partner violence (IPV) around the time of pregnancy is a recognized global health problem. Ethnic minorities and immigrant pregnant women experiencing IPV require culturally responsive health services. The aim of this scoping review was to identify aspects of cultural sensitivity in interventions to prevent or reduce IPV among ethnic minorities and immigrant pregnant women in high-income countries. Eight databases were searched in November 2019. Any type of scientific research, quantitative, qualitative, or mixed methods studies regarding interventions against IPV among pregnant women were considered for inclusion. Resnicow et al.’s definition of cultural sensitivity was used to identify aspects of cultural sensitivity. Ten papers relating to nine interventions/studies met our inclusion criteria. These studies, which included randomized controlled trials, a mixed methods study, a program evaluation, and a longitudinal study, were conducted in Australia, Belgium, Norway, and the United States. Aspects of surface cultural sensitivity, including the translation of intervention content into the language of the target group(s) and the involvement of bilingual staff to recruit participants, were identified in eight studies. Deep structure aspects of cultural sensitivity were identified in one study, where the intervention content was pretested among the target group(s). Results that could be related to the culture-sensitive adaptions included successful recruitment of the target population. Three studies were planning to investigate women’s experiences of interventions, but no publications were yet available. This scoping review provides evidence that culturally sensitive interventions to reduce or prevent IPV among immigrant pregnant women are limited in number and detail.

Intimate partner violence (IPV) is a global public health problem with societal and clinical implications for the women and men who are affected (Krug et al., 2002). The term “intimate partner violence” describes physical, emotional, and sexual violence, stalking, or psychological harm by a current or former partner (Garcia-Moreno et al., 2013). Worldwide, 30% of women have experienced physical and/or sexual IPV during their lifetime (Devries et al., 2013; Garcia-Moreno et al., 2013). Although IPV occurs in all cultures and across all social strata (Garcia-Moreno et al., 2013), women with low education and/or limited economic resources are at higher risk (Sanz-Barbero et al., 2019). Immigrant women and ethnic minority groups are likely to be overrepresented in these groups, hence be more exposed to IPV (Petrosky et al., 2017; Sanz-Barbero et al., 2019; Scheer et al., 2020). Both groups may face additional challenges such as cultural differences, isolation, prejudice, and racism (Gillum, 2009; Ogunsiji & Clisdell, 2017). In addition, immigrant women can experience language barriers and difficulties to navigate in the new country’s health services. (Lee & Hadeed, 2009; Leppala et al., 2020). There is evidence that both ethnic minorities and immigrant women underutilize health services for IPV (Hyman et al., 2009). These are aspects that need to be taken into consideration when interventions to reduce and prevent IPV are planned (Ogunsiji & Clisdell, 2017).

Pregnancy does not protect women from violence; rather, pregnancy can be a vulnerable period for women who are subject to IPV since this is a time of great change, including physical, emotional, social, and economic (Van Parys, Verhamme, et al., 2014). The prevalence of IPV in pregnancy ranges from 3% to 30% in different studies depending on the setting, measurements, and definitions (Devries et al., 2010; Finnbogadottir et al., 2014; James et al., 2013; Lukasse et al., 2014; Van Parys, Deschepper, et al., 2014). The majority of studies place prevalence in a range of 3.9%–8.7% (Devries et al., 2010).

Violence during pregnancy is associated with pregnancy complications and adverse outcomes such as premature contractions, miscarriage, premature birth, stillbirth, and low birth weight (Alhusen et al., 2014; Henriksen et al., 2013; Hill et al., 2016). Additionally, it may affect motherhood and the way women connect and interact with their babies (Hooker et al., 2016; Vatnar & Bjorkly, 2010).

Antenatal care is regarded as a “window of opportunity” to address IPV since women are in regular contact with health care professionals throughout the pregnancy (Devries et al., 2010), and routine inquiries regarding exposure to violence in antenatal care that have referral services are recommended (Garcia-Moreno et al., 2013). Van Parys, Verhamme, et al. (2014) conducted a systematic review of the effectiveness of interventions for IPV around the time of pregnancy and found that there is a lack of evidence regarding effective interventions. They recommended that the future focus should be on individual, relationships, community, and societal levels simultaneously. Cultural factors play a part across these levels and should be addressed in interventions that aim to reduce or prevent IPV. The differences in the prevalence of IPV during pregnancy between nations may also indicate that cultural factors influence IPV (Do et al., 2013).

There is consensus in the literature that there is a need for culturally responsive health services (Durieux-Paillard, 2011; Kreuter et al., 2003) and that interventions for members of minority populations have to be culturally sensitive to be tailored toward the needs of the population (Hölzel et al., 2016; Kreuter et al., 2003; Ogunsiji & Clisdell, 2017; Resnicow et al., 1999). Mainstream interventions usually target the majority population and fail to reach minority groups (Gillum, 2009; Ogunsiji & Clisdell, 2017). Studies show that culturally adapted programs support higher recruitment and program utilization (Perrino et al., 2018; Supplee et al., 2018). These interventions were available in women’s mother tongue and have involved experts of the target groups in the design of and recruitment for the studies. When different populations are engaged according to cultural understanding, it can result in more successful outcomes (Gillum, 2009; Resnicow et al., 1999).

Cultural sensitivity is defined as the extent to which ethnic and cultural characteristics, experiences, norms, values, behavioral patterns, and beliefs of a target population as well as relevant historical, environmental, and social forces are incorporated in the design, delivery, and evaluation of targeted health promotion materials and programs (Resnicow et al., 1999). Resnicow et al. (1999) described cultural sensitivity using two dimensions: surface structure and deep structure. Surface structure implies the matching of intervention materials and messages to the observable, “superficial” characteristics of a target population whereas deep structure pertains to the cultural, social, historical, environmental, and psychological forces that influence the target health behaviors of the population. When working with minority groups, factors such as religious beliefs, social networks, and traditional help-seeking behaviors have been identified as influential in how women respond to IPV (Bent-Goodley 2005; Fernandez, 2006). Hence, we consider Resnicows et al.’s model of culture sensitivity as an appropriate framework for evaluating culture-sensitive aspects in IPV interventions. Even though there is an agreement that health promotion programs should be culturally sensitive (Kreuter et al., 2003; Ogunsiji & Clisdell, 2017; Resnicow et al., 1999), there appears to be a lack of knowledge about how to address cultural differences in interventions aimed at preventing or reducing IPV during pregnancy. The main aim of this scoping review was to identify and present aspects of cultural sensitivity in interventions to prevent or reduce IPV among ethnic minorities and immigrant pregnant women in high-income countries. The specific research questions were the following:

1. What kind of culturally sensitive adaptations have been reported in interventions aimed at preventing or reducing IPV in pregnancy among ethnic minorities and immigrant women?
2. What are the results of the interventions and how are they related to culture-sensitive adaptions?
3. How do women experience these culturally sensitive interventions?

Accordingly, this scoping review provides an overview of the field, identifies and describes the methods used to develop and implement culturally appropriate interventions to reduce or prevent IPV against pregnant women. In addition, it aims to identify gaps in the research.

## Method

A scoping review is a methodology in which the existing literature is mapped, and research gaps are identified (Arksey & O’Malley, 2005). We consider the method to be beneficial in identifying available evidence and knowledge gaps in a given field (IPV and pregnancy) and to identify key characteristics and factors related to a specific concept (i.e., culture sensitivity; Munn et al., 2018). Scoping reviews are a transparent and thorough way to map and synthesize existing evidence (Arksey & O’Malley, 2005; Levac et al., 2010). In contrast to a systematic review with a meta-analysis, the quality of evidence is not evaluated in a scoping review, making the methodology time-efficient since the scope is usually broader (Levac et al., 2010). We followed the methods described in the Joanna Briggs Institute guidelines (Peters et al., 2015) and the steps described by Arksey and O’Malley (2005). We aimed to identify the aspects of cultural sensitivity described by Resnicow et al. (1999). Based on the definition of culture sensitivity as described above, the target groups of this scoping review were both immigrant women and women of ethnic minority groups. Detailed descriptions of Resnicow et al.’s surface and deep structure aspects of cultural sensitivity can be found in Supplementary Table S1. This scoping review is registered in the Open Science Framework.

### Search Strategy

Eight databases (MEDLINE, EMBASE, CINAHL, PsycINFO, Maternity & Infant Care Database, SocINDEX, Web of Science, and the Cochrane Library) were searched in November 2019 by a head librarian of our institution (EK). The full search strategy undertaken in MEDLINE is detailed in Table 1. The literature search was developed in collaboration between the head librarian (EK) and the first author (LH). Early in the process, it was decided that the search should consist of the following elements: (1) pregnant women and (2) IPV. The reason not to include key words related to culture sensitivity and interventions was our concern for eliminating relevant studies due to difficulty in capturing all possible key words. Thus, it was considered best to have a rather broad search including the concepts pregnant women and IPV and have the other elements as inclusion/exclusion criteria when screening the references. The amount of records identified with this broad search was considered acceptable to screen. This search strategy was translated for application to all the other databases with necessary adjustments. The search strategy in the other databases is available upon request. The librarian performed all searches. In addition, a search in Google Scholar, OpenGrey, and https://clinicaltrial.gov was performed, along with a broad search of full-text references, guidelines, and documents disseminated by relevant associations, societies, and institutions (i.e., World Health Organization, International Confederation of Midwives, and the Norwegian Midwifery Association). Finally, a citation search of the 10 included studies was performed in Google Scholar to identify other key articles. The search strategy was peer-reviewed by a university librarian. We updated the search in all databases in January 2021.

**Table 1.** MEDLINE Search Strategy.

#	Searches	Results
1	Pregnancy/	857,315
2	Pregnant Women/	7,757
3	perinatal care/ or prenatal care/	30,454
4	Midwifery/	18,770
5	(pregnan* or expectant mother*).tw, kw, kf.	507,188
6	(antenatal or prenatal or antepartum or perinatal).tw, kw, kf.	184,764
7	midwi*.tw, kw, kf.	24,222
8	or/1-7	1,040,631
9	Intimate Partner Violence/	2,032
10	Spouse Abuse/	7,295
11	Domestic Violence/	6,227
12	Gender-Based Violence/	143
13	(((partner* or spous* or husband* or wife or wives or marital or marriage* or married or domestic*) adj3 (abuse* or abusive* or violen* or victim* or battered or beat*)) or ipv).tw, kw, kf.	17,345
14	violence/ or physical abuse/	30,337
15	Exposure to Violence/	584
16	Battered Women/	2,607
17	((abuse* or abusive* or violen* or victim* or battered or beat*) adj3 (women* or woman* or wife or wives or pregnan* or expectant mother*)).tw, kw, kf.	10,823
18	or/14-17	41,016
19	Spouses/	9,731
20	(partner* or spous* or husband*).tw, kw, kf.	193,944
21	19 or 20	196,934
22	18 and 21	6,183
23	9 or 10 or 11 or 12 or 13 or 22	23,282
24	8 and 23	2,994
25	limit 24 to yr=“2000-Current”	2,546

### Inclusion and Exclusion Criteria

Scientific articles (quantitative, qualitative, and mixed methods studies) written in the languages the research team could read (Dutch, English, Norwegian, German, Swedish, or Turkish) that addressed interventions against IPV among pregnant women from high-income countries after the year 2000 were included. Studies without any culture-sensitive elements, as described in Supplementary Table S1, toward women of different ethnic backgrounds were excluded. Studies published prior to the year 2000 were excluded to ensure relevance to current practice. High-income countries (30 countries with the highest rates of income) defined by the Organization for Economic Co-operation and Development (2019) together with a combination of the Gender Inequality Index and Human Development Index were chosen (United Nations Development Programme, 2019; Supplementary Table S2).

### Data Extraction, Synthesis, and Analysis

In the initial screening, all the search results were imported into reference management software (EndNote version 19), and duplicates were removed by the librarian (EK). All the titles and abstracts were uploaded into Rayyan QCRI and assessed independently by two researchers. The same procedure was followed during the updated search in January 2021. The full-text versions of the papers that met the inclusion criteria were retrieved and assessed for eligibility by three teams comprising two reviewers each, who reviewed the papers independently. An extraction form was developed and used for each paper. The extraction form included data about the title of the paper, study aims, population, sample size, concept, study setting, cultural sensitivity elements, and women’s experiences. Potential conflicts were solved by a third reviewer (LH). Additional papers found by searching the reference lists of the included papers, and other sources were assessed as described above. One reviewer (LGH) extracted the study characteristics and findings and entered them into a customized table (Table 2).

## Results

**Figure 1.**
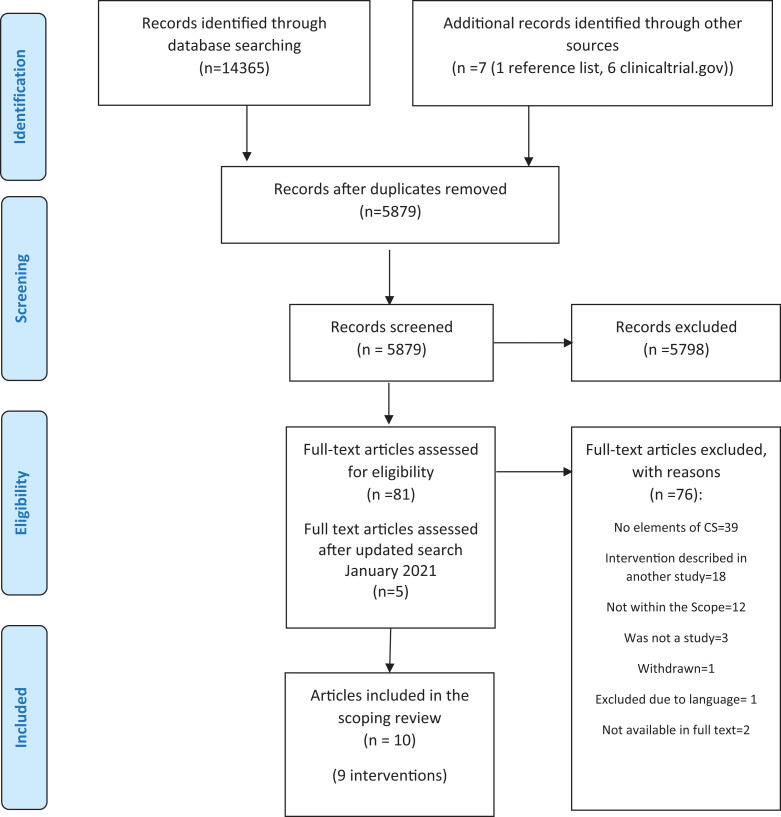
PRISMA flowchart.

In total, 14,365 citations were identified. One additional citation was identified from the search of the articles’ reference lists and six from a search of https://clinicaltrial.gov, leaving 5,879 records to be screened after duplicates were removed. The full text of 81 papers was initially assessed. An updated search in January 2021 identified five full-text papers leaving the total number of full-text articles to be 86, of which 76 were excluded based on the inclusion/exclusion criteria. Supplementary Table S3 gives an overview of the excluded papers with reason. In total, 10 articles describing nine interventions were included in this study. The citation search of the references of the included studies did not yield any additional studies. The flow of the study selection process is shown in a Preferred Reporting Items for Systematic reviews and Meta-Analyses (PRISMA) flowchart (Figure 1; Moher et al., 2015).

### Characteristics of the Included Studies

Of the 10 included papers, which related to nine interventions/studies (see Table 2), six were from the United States, one from the Netherlands, one from Australia, and one from Norway. The study designs included randomized controlled trials (RCT) (*n* = 6; Feder et al., 2018; Henriksen et al., 2019; Johnson et al., 2020; Katz et al., 2008; Langhinrichsen-Rohling & Turner, 2012; Taft et al., 2009; Taft et al., 2011), a program evaluation (*n* = 1; Kramer et al., 2012), a longitudinal study (*n* = 1; McFarlane et al., 2000), and a qualitative and quantitative mixed methods study that included an observational study with pre- and posttests (*n* = 1; Loeffen et al., 2011). The study sample sizes ranged from 21 (Loeffen et al., 2011) to 1,044 (Katz et al., 2008). Most of the participants were recruited through prenatal care, home care visitations, or nurse–family partnerships.

**Table 2.** An Overview of the Studies With a Summary of the Results.

Author (Year), Location	Study Participants	Methods Name of the Study	Description of the Intervention	Study Outcome	Culture-Sensitive Adaptations	Results Related to the Culture-Sensitive Adaption
Feder et al. (2018), United States	Pregnant women <28 weeks of pregnancy (*n* = 238)	RCT	The intervention contained three main components: nurse training and screening assessment of intimate partner violence (IPV), a secondary prevention component for those reporting IPV, and a primary prevention component for all participants.	Positive results regarding preventing some form of IPV but not reducing violence among those who experienced IPV at the beginning of the study	Surface structure	Successful in recruiting target population
Henriksen et al. (2019), Norway	Pregnant women ≥18 years at any gestational age who understand Norwegian, Urdu, Somali, or English	RCT The safe pregnancy study Protocol paper	A study conducted in antenatal care. Women who screen positive on violence questions via a tablet were randomized to an intervention video containing information about violence and safety behaviors or a control video with information on having containing information about violence and safety behaviors or a control video with information on having a safe pregnancy in general.	Protocol paper. No results are published	Surface and deep structure	Protocol paper. No results published
Johnson et al. (2020), United States	Pregnant women ≥18–45 years seeking help at a mental health clinic	RCT Strength for U in Relationship Empowerment Protocol paper	Brief, interactive program targeting IPV that consists of motivational interviewing and incorporates empowerment strategies.	Protocol paper. No results published	Surface structure	Protocol paper. No results published
Katz et al. (2008), United States	Self-identification as Black/African American or Latina (*n* = 1,044), residence in the District of Columbia, ≥18 years of age, English speaking	RCT DC-HOPE	A 10-session intervention delivered by counselors at pre- and postpartum care visits. A brochure provided information about different types of violence, danger assessment, and preventive options women might consider allowing an individualized focus on areas of need for that woman at each intervention visit.	The intervention content was likely to be covered by the participants, and 68% of the sessions regarding IPV were covered fully or partially	Surface structure	Successful in recruiting target population
Kramer et al. (2012), United States	Pregnant and newly delivered women (*n* = 373) who self-disclose IPV during screening in health care settings and the community	Program evaluation Safe Mom, Safe Baby (SMSB)	A nurse-led interdisciplinary clinical program for pregnant and recently delivered clients is experiencing IPV. Approximately half the women received intensive and frequent contact (two to three times per week for months). The women in the intervention group should develop a personal safety plan.	They measured readiness for change and indicated a positive effect among those receiving the intervention	Surface structure	Successful in recruiting target population
Langhinrichsen-Rohling & Turner (2012), United States	Inner-city adolescent girls (*n* = 72) who were receiving teen pregnancy services. The majority of participants identified themselves as African American (93.1%); the remaining identified themselves as Caucasian (4.2%) or other (2.8%)	RCT Building a Lasting Love (BALL)	BALL was designed to teach healthy relationship skills to high-risk adolescent girls who were pregnant. The BALL curriculum consists of four sessions, each lasting an hour and a half, administered once per week so that the entire intervention could be administered in 1 month.	At the end of the BALL project, a lower percentage of the women in the intervention group reported severe IPV	Surface structure	Successful in recruiting target population
Loeffen et al. (2011), the Netherlands	Mothers with home living children or pregnant women who are victims of IPV	Mixed methods qualitative and quantitative, including an observational study with pre- and posttests	Mentor mother support by the participating family physicians. Women in the intervention arm received up to 4 months’ support from trained and supported nonprofessional mentor mothers. The mentor mother support consisted of a 1-hr weekly visit by the mentor mother. At each visit, the mentor mother provided nonjudgmental active listening and support to develop a trusting relationship with the woman.	The study by Loeffen et al. is a protocol paper. Paper reporting result did not include pregnant women but mothers in general (Loeffen et al., 2017)	Surface structure	Successful in recruiting a diverse sample (Loeffen et al., 2017)
McFarlane et al. (2000), United States	Pregnant, physically abused Hispanic women (*n* = 329)	Longitudinal study	Women were randomized to one of the three interventions: Brief, Counsel, or Outreach. The brief intervention consisted of providing a wallet-sized resource card that included phone numbers of local agencies to assist with domestic violence and information about planning for personal safety. The counseling intervention consisted of unlimited access to counseling services of a female, bilingual Spanish-speaking, professional counselor with expertise in domestic violence. The outreach intervention consisted of the same unlimited access to the professional counselor plus the services of a mentor mother.	Reported significantly lower violence scores in their intervention groups	Surface structure	Partly successful in recruiting target population
Taft et al. (2009), Australia Taft et al.(2011), Australia	Women who are pregnant or with infants, abused, or symptomatic of abuse, 206 in the intervention and 112 in the comparison arm	Cluster RCT (protocol paper and sample description) MOSAIC	Women in the intervention arm received up to 12 months of support from trained and supported nonprofessional mentor mothers. For women identified as abused or at risk of abuse, the MOSAIC mentor mothers provided regular (on average, weekly) contact and support through phone calls, home visits, and outings; assisted in developing safety strategies; and provided information about and assisted with resources and referrals to community services. The study was designed to be inclusive for Vietnamese women in addition to English-speaking women.	Reported significantly lower violence scores in their intervention groups	Surface structure	Recruited a small sample of Vietnamese women

### Description of the Interventions

All the included studies planned/provided interventions and details of the interventions are described in Table 2. The majority consisted of different counseling sessions delivered by health professionals/trained counselors (Feder et al., 2018; Katz et al., 2008; Kramer et al., 2012; Langhinrichsen-Rohling & Turner, 2012; McFarlane et al., 2000). The counseling differed from unlimited access to counseling services during pregnancy (McFarlane et al., 2000) to four short sessions during 1 month (Langhinrichsen-Rohlingand & Turner, 2012). In three studies, the counseling was delivered as part of home visitation programs (Feder et al., 2018; Katz et al., 2008; Kramer et al., 2012). In two studies, the intervention was computerized (Henriksen et al., 2019; Johnson et al., 2020). Henriksen et al. used an information video (Henriksen et al., 2019) and Johnsen et al. aiming to use an interactive program consistent with motivating interviewing (Johnson et al., 2020). Two studies had mentor mothers supporting the participants (Loeffen et al., 2011; Taft et al., 2009; Taft et al., 2011). Three of the interventions targeted IPV prevention (Feder et al., 2018; Katz et al., 2008; Langhinrichsen-Rohling & Turner, 2012) and six aimed at reducing IPV (Henriksen et al., 2019; Johnson et al., 2020; Kramer et al., 2012; Loeffen et al., 2011; McFarlane et al., 2000; Taft et al., 2009; Taft et al., 2011). The study’s main findings are described in Table 2.

### Identified Aspects of Cultural Sensitivity

Elements of surface cultural sensitivity were identified in all studies (Feder et al., 2018; Henriksen et al., 2019; Johnson et al., 2020; Katz et al., 2008; Langhinrichsen-Rohling & Turner, 2012; Loeffen et al., 2011; McFarlane et al., 2000; Taft et al., 2009; Taft et al., 2011). In the Feder et al. (2018) study, the intervention could be delivered in Spanish by Spanish-speaking nurses if preferred. In the Safe Pregnancy study, the questionnaires, information sheets, and intervention content were available in Norwegian, Somali, Urdu, and English. Focus groups were conducted at a crisis shelter for a user-involvement study to review the intervention with an expert community (Flaathen et al., 2020). The study by Johnson et al. (2020) describes how they included women from the target population in focus groups to ensure a broadly applicable intervention. They used images of different ethnic groups to include a range of women. The pregnancy advisors providing the intervention in the Healthy Outcomes of Pregnancy Education (DC-HOPE) study were African American or Hispanic and had experience in counseling minority populations (Katz et al., 2008). In the Safe Mom, Safe Baby (SMSB) study, bicultural and bilingual staff were involved to provide insights into the diverse needs of the target group (Kramer et al., 2012), while in Langhinrichsen-Rohling and Turner’s (2012) study, the survey was read aloud to facilitate comprehension and to alleviate the concerns of participants who were poor readers. In Loeffen et al.’s (2011) study, the mentor mothers who delivered the intervention were selected based on their cultural backgrounds. In the intervention, the cultural preferences of the abused mothers were taken into account when matching them with the mentor mothers (Loeffen et al., 2011). The mentors in McFarlane’s (2000) study were bilingual Spanish-speaking women who were mothers themselves and resided in the communities served by the prenatal clinics. The intervention material was also available in Spanish (McFarlane et al., 2000). In the MOthers’ Advocates In the Community (MOSAIC) study (Taft et al., 2009; Taft et al., 2011), Vietnamese women and Vietnamese GPs recruited Vietnamese women. Vietnamese radio was also used to publicize the study. Vietnamese mentors were selected to support the Vietnamese women during the intervention. Some of the mentors were refugees, immigrants, or themselves survivors of violence, and the mentor training included cross-cultural understandings of IPV. The survey instrument was first translated into Vietnamese before being translated back into English. The focus group was then assessed by Vietnamese psychologists and bilingual family violence workers (Taft et al., 2009; Taft et al., 2011).

Aspects of deep structure cultural sensitivity were only identified in one study (Henriksen et al., 2019). Flaathen et al. (2020) conducted a user-involvement study to pretest the content of the intervention video and questionnaire among Somali and Pakistani women as well as professionals with expert knowledge of the target groups.

### Results Related to Culture-Sensitive Adaptions

The elements of surface structure cultural sensitivity identified in the included studies were related to translating the content into different languages and having bicultural and bilingual staff delivering the intervention. Five of the included studies reported whether the targeted population was recruited (Feder et al., 2018; Katz et al., 2008; Langhinrichsen-Rohling & Turner, 2012; McFarlane et al., 2000; Taft et al., 2009). In the study by Feder et al., approximately 50% was African American/Hispanic/Latina in the intervention group and more than 60% in the control group. Katz et al. (2008) only recruited African American women and the majority of the participants in the study by Langhinrichsen-Rohling and Turner’s were African American. McFarlane et al. (2000) recruited 96% Hispanic women. In the study by Taft et al. (2009), designed to reach Vietnamese women in addition to the general population, 17% were Vietnamese in the intervention group and 7% in the control group (Taft et al., 2009).

The one study, in which surface and deep structure elements were included, is a protocol paper and results are not published (Henriksen et al., 2019). No studies reported results, other than the recruited population, that could be related to the culture-sensitive adaptions.

### Women’s Experiences of the Interventions

In total, three studies aimed to investigate the participants’ experiences of the interventions (Henriksen et al., 2019; Kramer et al., 2012; Taft et al., 2009; Taft et al., 2011). In the Safe Pregnancy study, the women’s experiences of the study will be investigated using qualitative interviews (Henriksen et al., 2019). As part of the process improvement program in the SMSB study, 13 women were interviewed about their experiences (Kramer et al., 2012). Taft et al. (2009, 2011) conducted telephone interviews with 11 Vietnamese women and four Vietnamese mentors to explore their overall experiences of mentoring and being mentored. However, contact with the project leader of this study revealed that the results of this study have not yet been published.

## Discussion

This scoping review revealed few culturally sensitive adaptations in interventions aiming to prevent or reduce IPV in pregnancy. The adaptions identified were mainly based on surface structure cultural sensitivity. One study delivered deep structure culturally sensitive elements. Given the prevalence of IPV among ethnic minorities and women with immigrant backgrounds in high-income countries (Petrosky et al., 2017; Sanz-Barbero et al., 2019; Scheer et al., 2020), there is an urgent need for more knowledge on how to tailor interventions toward the needs of these populations. There may be several reasons for this lack of culturally sensitive adaptations.

First, culturally sensitive adaptations imply additional effort and costs for researchers (George et al., 2014). As most study samples reflect the population, most of the participants in such studies would include nonimmigrants. Including immigrant groups in a study would therefore require extra interest and commitment and strong motivation for doing so. The most eminent aspects of cultural sensitivity in the studies included in this review related to the surface structure. Surface structure adaptations of cultural sensitivity suggest the matching of intervention materials and messages to the observable, “superficial” characteristics of a target population(s) (Resnicow et al., 1999). In the included studies, the intervention content and instruments were often translated into the language of the target group (Feder et al., 2018; Henriksen et al., 2019; Langhinrichsen-Rohling & Turner, 2012; McFarlane et al., 2000; Taft et al., 2009; Taft et al., 2011). The researchers also used bilingual and bicultural staff to recruit and deliver their interventions (Feder et al., 2018; Katz et al., 2008; McFarlane et al., 2000; Taft et al., 2009; Taft et al., 2011). Results related to these culture-sensitive adaptions included successful recruitment of the target population in the majority of the included studies, hence this may be viewed as successful adaptions. Other studies support that these examples of cultural adaption have a positive effect on participant engagement (Perrino et al., 2018; Supplee et al., 2018). This kind of cultural sensitivity is the easiest to achieve in study settings that are already challenging (Resnicow et al., 1999).

Deep structure cultural sensitivity requires an understanding of the core cultural elements, including the social, cultural, historical, environmental, and psychological forces that influence the target health behaviors in the proposed target population(s) (Resnicow et al., 1999). In the present scoping review, aspects of deep structure cultural sensitivity were identified in only one study (Henriksen et al., 2019). We did not find any studies that reported having the intention to perform—or that had performed—focus group interviews with the target population(s) to investigate how religion, family, society, economics, and government influence their coping strategies and stressors with respect to IPV. These applications require adequate research funding and the availability of researchers with immigrant backgrounds (George et al., 2014). Alternatively, it may be perceived as stigmatizing to select certain immigrant groups for studies on IPV, and this is therefore avoided, especially in superficial culturally sensitive interventions.

Deep culturally sensitive interventions require close collaboration with the targeted communities as “experts” and collaborators in the adaptation (Lyon et al., 2017; Okamoto et al., 2014). This level requires locating members of the target population with sufficient knowledge of the main population’s social and legal norms, and preferably language, while still being in firm contact with their original cultural backgrounds (Resnicow et al., 1999). The Mothers in Motion study applied deep structure components in a community-based intervention to prevent weight gain among low-income African American women (Chang et al., 2014). The researchers worked closely with the community and peer advisory groups when planning and evaluating the intervention. The intervention had positive outcomes on the participants’ self-efficacy to cope with stress (Chang et al., 2019).

We only found three studies expressing the intent to conduct qualitative interviews to investigate women’s experiences of the interventions. However, none of the studies have yet reported their results (Henriksen et al., 2019; Kramer et al., 2012; Taft et al., 2009; Taft et al., 2011). This is expected given the limited number of interventions that include deep structure cultural sensitivity. Qualitative approaches can contribute in several ways to the development and evaluation of complex health interventions (Lewin et al., 2009). The results of this review support the need to conduct further research to discuss the experiences of women who have participated in culturally sensitive interventions against IPV during pregnancy.

The challenges we had identifying surface culture adaptations in some studies may have been because the studies did not describe the culturally sensitive aspects in detail. For instance, in the SMSB study (Kramer et al., 2012), the participants drove the development of their personal safety plans. They identified their readiness to engage in various service options, which may have included crisis intervention, emotional support, advocacy within various health care and community systems, and assistance with specific safety strategies. This individualized approach would very likely have allowed for both surface- and deep-level structures of culture sensitivity. However, it was not possible to identify whether this individualized approach actually achieved this. In the DC-HOPE study, the intervention was built on a conceptual framework that posited the interactive role of the individual and the social environment (Katz et al., 2008). However, the study did not provide details on how this framework was applied. The use of illustrations of women representing the target group in the brochure was another important surface cultural sensitivity application that was difficult to investigate in this scoping review because it was not commented upon in the description of the recruitment process.

## Conclusions

This study highlights the gaps in the literature regarding cultural sensitivity interventions to reduce or prevent violence against pregnant women. Based on the findings of this study, we concluded that:

- There is a lack of culturally sensitive adaptations reported in interventions aiming to prevent or reduce IPV in pregnancy among immigrant and ethnic minority women.
- Translation of the intervention content to the language of the target group as well as the presence of bilingual and bicultural staff to recruit and deliver the intervention were the most eminent surface structure adaptations of cultural sensitivity.
- None of the included studies investigated the influence of cultural, social, historical, environmental, or psychological forces on the target health behaviors in the target populations.
- None of the studies provided feedback on the experiences of the women participating in the interventions against IPV during pregnancy

### Implications for Research and Practice

- Health care professionals should be aware of aspects of cultural sensitivity when they communicate with pregnant women.
- The development of interventions to prevent IPV against immigrant and ethnic minority pregnant women should include people who are experts in IPV or who have experience in crisis shelters and should consult with the target group(s) regarding the intervention development process.
- Further studies are needed to examine aspects of culturally sensitive interventions and women’s experiences of deep structure culturally sensitive elements and to measure the effectiveness of existing culturally sensitive interventions in preventing violence against immigrant and ethnic minority pregnant women.

### Strengths and Limitations of the Study

To our knowledge, this is the first study to provide evidence that culturally sensitive interventions to reduce or prevent violence against immigrant and ethnic minority pregnant women are limited in number and detail. However, there were some limitations to this scoping review. First, we applied Resnicow’s definition of surface and deep structure cultural sensitivity to investigate the interventions. Although this model has been widely applied in the development of culturally sensitive programs related to healthy eating as well as cancer and stroke prevention, it has not previously been applied to interventions aiming to prevent or reduce IPV. It has to be mentioned that the use of other frameworks may have led to different outcomes. Second, studies not describing aspects of cultural sensitivity, which have included substantial numbers of pregnant women of different ethnic backgrounds, were excluded (Humphreys et al., 2011; Sharps et al., 2016). Third, this scoping review was restricted to articles published after the year 2000. Culturally sensitive interventions before the year 2000 were therefore not captured in this review. Despite these limitations, we believe that the results of this scoping review may have implications for the future use of culturally sensitive interventions to prevent or reduce violence against pregnant women with ethnic backgrounds.

## Supplemental Material

Supplemental Material, sj-pdf-1-tva-10.1177_15248380211021788 - Cultural Sensitivity in Interventions Aiming to Reduce or Prevent Intimate Partner Violence During Pregnancy: A Scoping Review Click here for additional data file. Supplemental Material, sj-pdf-1-tva-10.1177_15248380211021788 for Cultural Sensitivity in Interventions Aiming to Reduce or Prevent Intimate Partner Violence During Pregnancy: A Scoping Review by Lena Henriksen, Sezer Kisa, Mirjam Lukasse, Eva Marie Flaathen, Berit Mortensen, Elisabeth Karlsen and Lisa Garnweidner-Holme in Trauma, Violence, & Abuse
