# Supplementary material for: Cultural Sensitivity in Interventions Aiming to Reduce or Prevent Intimate Partner Violence During Pregnancy: A Scoping Review
Source: Trauma Violence Abuse. 2021 Jun 10;24(1):97–109. doi: 10.1177/15248380211021788 (PMC9660282; doi:10.1177/15248380211021788)
Supplement: Supplemental Material, sj-pdf-1-tva-10.1177_15248380211021788 - Cultural Sensitivity in Interventions Aiming to Reduce or Prevent Intimate Partner Violence During Pregnancy: A Scoping Review [file sj-pdf-1-tva-10.1177_15248380211021788.pdf]

Supplementary table 1: Surface and deep structure cultural sensitivity according to Resnicow et al. (Resnicow et al., 1999).

**Surface structure cultural sensitivity** means to which extent do the interventions meet the target population where they are; how well do they fit within their culture and their experience. Surface structure CS establishes feasibility of the intervention.		
**Surface structure CS**	**Further explained**	**Examples**
Matching intervention materials and messages to observable, "superficial" characteristics of a target population	This may involve using people, places, language, music, food, product brands, locations and clothing familiar to, and preferred by the target audience.	The questions are translated to the language(s) of the target group(s). Uses staff with knowledge about IPV, staff with cultural knowledge.
Uses channels (e,g. media) and settings (e.g hospital, community health centres) that are most appropriate for delivery of messages and programs		Using settings where women are safe and feel safe, not with their partner.
Expert and community review of the intervention	Does the study include experts within the field to assess the intervention	Uses people who are experts or have experience within the field of IPV, e.g staff from crisis shelters
Involvement of the target population in the intervention development process	Has the target group been involved in the process of developing the intervention?	Discussing the intervention development process with women who have experienced IPV or any other involvement
Focus groups discussion including surface structure elements	Has the researchers conducted focus groups to gain target groups perceptions or experiences of surface elements in the intervention?	Gaining target groups perceptions of surface structure elements as mentioned above. E.g using pictures that shows other ethnicities receiving the intervention (a Pakistani using safety behaviours)
Pretesting the material to members of the target population to obtain feedback regarding format and content	Has the intervention content and questionnaires been discussed with the target population?	

**Deep structure CS:** requires understanding of the social, cultural, historical, environmental and psychological forces that influence the target health behaviour in the proposed target population. Determines program impact.		
Investigate how religion, family, society, economics, and government influence the target behaviour	Have the researchers conducted in-depth interviews (IDIs) or focus groups (FGs) with target population to investigate the factors that influence the target behaviour? E.g how IPV are perceived from religion or society	To achieve CS it is essential to assume significant heterogeneity of the target population. There will be variability within the group which is important to investigate.

Explore stressors and coping strategies regarding the health behaviour, of the target population	Have the researchers conducted IDIs or FGs with the target population to explore their coping strategies and stressors regarding IPV?	
Pretesting the material to members of the target population to obtain feedback regarding format and content	Has the material been discussed with the target population?	

Supplementary table 2: Included studies: World Bank, "OECD High-Income countries"
in combination with top 30 in the “Gender Inequality Index” and “Human Development Index”.

	Countries from which studies will be included
1	Austria
2	Belgium
3	Czech Republic
4	Denmark
5	Estonia
6	Finland
7	France
8	Germany
9	Greece
10	Hungary
11	Iceland
12	Ireland
13	Italy
14	Latvia
15	Lithuania
16	Luxembourg
17	Netherlands
18	Norway
19	Poland
20	Portugal
21	Slovakia
22	Slovenia
23	Spain
24	Sweden
25	Switzerland
26	United Kingdom
27	Israel
28	Japan
29	South Korea
30	Canada
31	United States
32	Australia
33	New Zealand

Supplementary table 3. Overview over excluded studies with reason

	**Author, country (year)**	**Excluded – reason**
1	Bacchus, L. US (from clinicaltrial.cov) (2012)	Described in Bacchus et al 2016
2	Bacchus, L. J., et al. US (2016a)	No elements or aspects of CS.
3	Bacchus, L. J., et al. US (2016).	No elements or aspects of CS.
4	Backonja, U., et al. US (2016).	Described in Kiely, M., et al. (2010).
5	Barlow et. Al US (2007)	No elements or aspects of CS.
6	Bloom, T. L., et al.US (2014).	No elements or aspects of CS.
7	Burnett, C., et al. US (2019)	No elements or aspects of CS.
8	Calderon, S. (from clinicaltrial.cov) US 2007	Described in Calderon, S. H., et al. (2008)
9	Calderon, S. H., et al. US (2008)	No elements or aspects of CS.
10	Catherine, N. L., et al. Canada (2016).	No elements or aspects of CS.
11	Chen, Y & Olson-Chen US (2020	No elements or aspects of CS.

12	Coker, A. L., et al. US (2012)	No elements or aspects of CS.
13	Cunningham, S.D. (from clinicaltrial.cov) US 2017.	Not within the scope.
14	Curry, M. A., et al US (2006).	No elements or aspects of CS.
15	Daoud, N. Israel (2020)	Not within the scope.
16	Dauber, S., et al. US (2017)	Not within the scope.
17	Dauber, S., et al. US (2020)	Not within the scope.
18	Domoney, J., et al. UK (2019)	No elements or aspects of CS.
19	Eddy, T., et al. US (2008)	No elements or aspects of CS.
20	El-Khorazaty, M. N., et al. US (2007)	Described in Kiely, M., et al. (2010).
21	El-Mohandes, A. A., et al. (2008)	Described in Kiely, M., et al. (2010).
22	Joseph, J. G., et al. US (2009)	Described in Kiely, M., et al. (2010).
23	Feder, L. (from clinicaltrial.cov) 2013	Described in Federer et al 2018.
24	Florsheim, P., et al. US (2011)	No elements or aspects of CS.
25	Florsheim, P., et al. US (2019)	No elements or aspects of CS.
26	Gance-Cleveland, B., et al. UK (2019)	No elements or aspects of CS.
27	Gerbert, B. Clinicaltrial.gov US (2007).	Described in Humphreys, J., et al. (2011)
28	Gunn, J., et al. Australia (2006)	Not within the scope.
29	Hegarty, K., et al. Australia (2007).	Not within the scope.
30	Herrman, J., et al. US (2018)	No elements or aspects of CS.
31	Heyman, R. E., et al. (2019).	Not within the scope.
32	Humphreys, J., et al. US (2011)	No elements or aspects of CS.
33	Jack, S. M., et al. US (2019).	No elements or aspects of CS
34	Jacobs, F., et al. US (2016).	Not within the scope.
35	Janssen, P. Clinicaltrial.gov Canada 2009	The study was withdrawn.
36	Johnson, J. Clinicaltrial.gov (2017)	Protocol – no study yet. No aspects of CS
37	Kan, M.L et al US (2021)	No elements or aspects of CS.
38	Kastello, J. C., et al. US (2016)	Described in Sharps et al (2016)
39	Kataoka, Y., et al. (2010)	Not available in full text.
40	Kemp, L., et al. Australia (2006).	No elements or aspects of CS.
41	Kiely, M., et al. US (2010).	Described in Katz et al., 2008
42	Macdonald, G., et al. UK (2018)	No elements or aspects of CS.
43	McDonal, G. Isrctn UK (2017).	Described in Macdonald, G., et al. UK (2018)
44	McFarlane, E., et al. US (2013)	Not within the scope.
45	McNutt, A.L, Ford, D. US Clinicaltrial.gov (2005)	Not within the scope.
46	Mejdoubi, J., et al. The Nederlands (2011)	Culturally adapted to Dutch society, but not to ethnic minorities, hence no elements of CS as described in this Scoping Review.
47	Mejdoubi, J., et al. The Nederlands (2013)	Described in Mejdoubi, J., et al. (2011).
48	Memorial hospital, US Clinicaltrial.gov 2008	Described in Zlotnic et al 2008.
49	Michalopoulos, C. US, Clinicaltrial.gov 2014 (MIHOPE)	No elements or aspects of CS.
50	Miller-Graff, L Clinicaltrial.gov (2019)	Protocol – no study yet. No aspects of CS
51	Miyazaki, C. and Y. Kataoka (2010)	In Japanese
52	Nagle, G. A. US (2003)	No elements or aspects of CS.
53	Olds, DL. Et al., US (2004)	No elements or aspects of CS.
54	Paruchuri, Y., et al. US (2018)	No elements or aspects of CS.
55	Petersen, R., et al. US, (2001)	Not within the scope.
56	Price, S. K., et al. US, (2017)	No elements or aspects of CS.
57	Reading, R. US, (2005).	Described in Olds, DL. Et al., US (2004).
58	Reading, R. US (2007).	Described in Barlow et al 2007.
59	Sharps, P. W., et al. US (2016)	No elements or aspects of CS.
60	Sharps, P., et al. US (2013)	No elements or aspects of CS.
61	Steen, M., et al. US (2010)	No elements or aspects of CS.
62	Stubbs, J. M. and H. M. Australia (2012)	No elements or aspects of CS.
63	Subramanian, S., et al. US (2012)	No elements or aspects of CS.

64	Taft, A., et al. (2003).	Conferences abstract. Has been requested from library, not available.
65	Taft, A. J., et al. Australia (2012)	Described in Taft A.J. 2015.
66	Taft, A. J., et al. Australia (2015)	No elements or aspects of CS.
67	Tandon, D., et al. US (2005)	Not within the scope
68	Tandon, D., et al. US (2019)	No elements or aspects of CS.
69	Van Parys Clinicaltrial.gov Belgium (2010)	Described in Van Parys et al., (2017).
70	Van Parys, A. S., et al. Belgium (2017).	No elements or aspects of CS.
71	Woodford, M. US Clinicaltrial.gov (2017)	No elements or aspects of CS.
72	Zlotnick et al Clinicaltrial.gov US (2008)	No elements or aspect of CS
73	Zlotnick, C Clinicaltrial.gov US (2015)	Described in Zlotnic et al.2019
74	Zlotnick, C., et al. US (2011)	No elements or aspects of CS.
75	Zlotnick, C., et al. US (2019)	No elements or aspects of CS.
76	Zlotnick, C US Clinicaltrial.gov (2020)	Protocol - described in Johnson 2020
